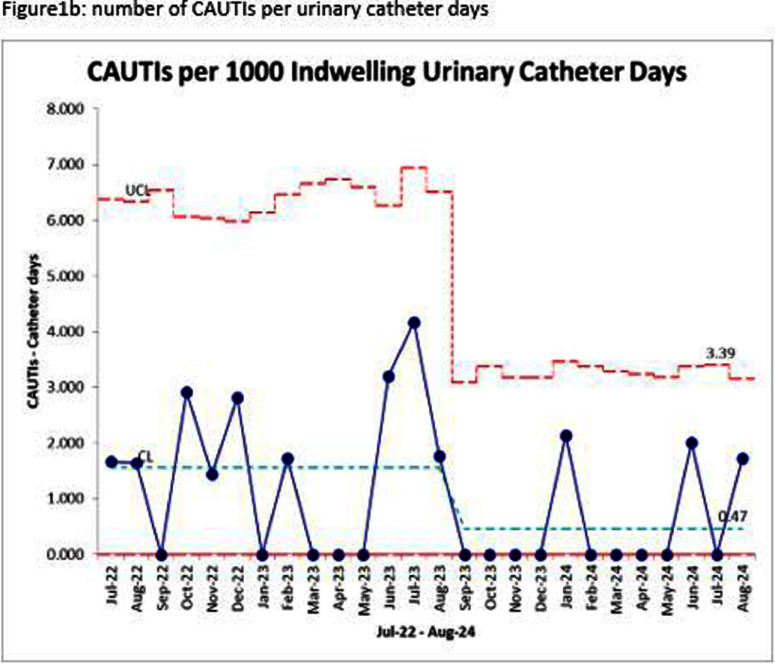# Optimizing Diagnostic Stewardship: Reducing CAUTI Rates Through Urine Culture Decision-Making in the ICU

**DOI:** 10.1017/ash.2025.282

**Published:** 2025-09-24

**Authors:** Nicole Wiltfang, Karen Brust, Oluchi Abosi, Takaaki Kobayashi, Elizabeth Krigbaum

**Affiliations:** 1 University of Iowa Health Care; 2 University of Iowa Health Care; 3 University of Iowa Hospital and Clinics; 4 University of Kentucky; 5 University of Iowa Healthcare

## Abstract

**Background:** Approximately half of all fevers in intensive care units (ICUs) are attributed to noninfectious causes. Despite this, most providers routinely culture urine from patients with indwelling urinary catheters who develop a new fever, which can lead to overdiagnosis and unnecessary antibiotic use. This study evaluated the impact of transitioning from a urinalysis (UA) with reflex to culture order to a stand-alone UA with microscopy in the Surgical and Neurosciences Intensive Care Unit (SNICU) on the frequency of urine cultures ordered and Catheter-Associated Urinary Tract Infections (CAUTIs). **Methods:** This quasi-experimental before-and-after study was conducted at the University of Iowa between July 2022 and August 2024 and included all SNICU patients. In August 2023, SNICU staff were educated to send a UA with microscopy, review results with the care team, and then decide whether a reflex to culture was warranted. This initiative was collaboratively developed by SNICU leadership and the hospital epidemiology team. Data on the frequency of urine cultures and CAUTI rates per 1,000 catheter days were compared before and after implementation using a P chart in QI Macros. **Results:** During the pre-intervention period, SNICU ordered approximately 66 urine cultures per 1,000 patient days, with a CAUTI rate of 1.55 per 1,000 catheter days (Figure 1a and 1b). While all data points remained within control limits, red data points between November 2022 and January 2023 indicated possible special cause variation; after further investigation, the specific cause was not identified and data points returned to normal cause variation. Following implementation, the frequency of urine cultures decreased to approximately 32 per 1,000 patient days, and the CAUTI rate dropped to 0.47 per 1,000 catheter days. The intervention also resulted in greater process stability, as evidenced by a narrower range between the upper control limit (48.97) and lower control limit (15.36). These improvements demonstrated the effectiveness of transitioning to a deliberate, decision-making process based on UA with microscopy. **Conclusion:** Transitioning from reflex urine culture orders to a stand-alone UA with microscopy, combined with provider decision-making and leadership engagement, significantly reduced the frequency of urine cultures and CAUTI rates in the SNICU. By requiring a deliberate review of UA results before ordering cultures, this intervention successfully optimized diagnostic stewardship. The pilot program will be integrated into the electronic medical record and expanded to other units.

**Figure:**
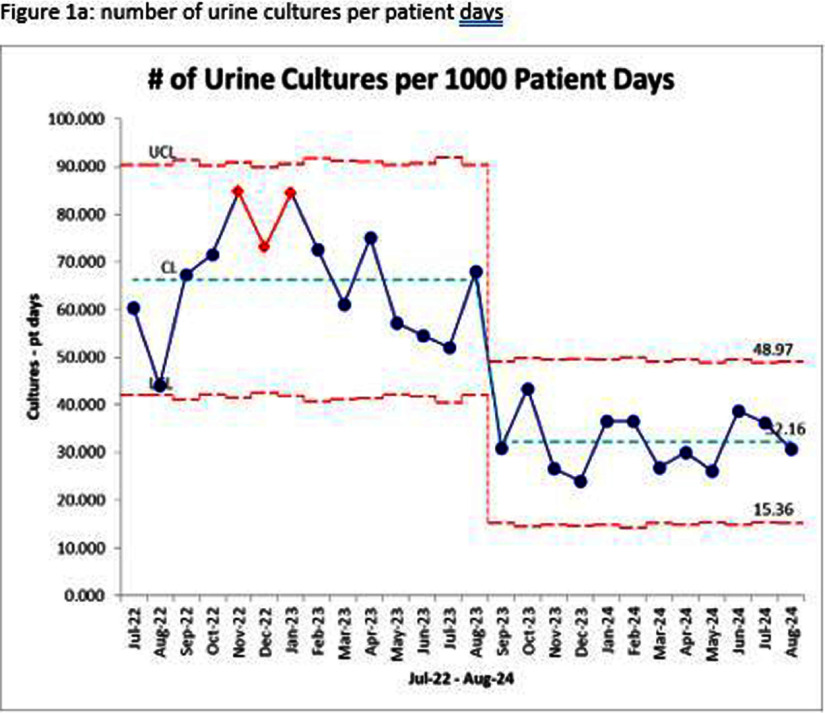

**Figure:**